# Visualization analysis of research progress for walkability

**DOI:** 10.1038/s41598-024-52227-9

**Published:** 2024-01-23

**Authors:** Xuan Li, Dan Xie, Zhiyu Zhou, Xin Zhang, Rui Li, Jiayi Li, Zeyu Chen, Jiayu Zhang

**Affiliations:** 1 School of Architecture & Art Design, Hebei University of Technology, Tianjin, 300401 China; 2 Key Laboratory of Healthy Habitat Environment in Hebei Province, Tianjin, 300401 China

**Keywords:** Information technology, Scientific data

## Abstract

The study of walkability is of great significance to the construction of healthy cities. In this paper, taking 1283 articles of walkability, which were included in Web of Science, as the research object. This paper adopts to analysis the research progress by using the method of scientometrics and knowledge networks analysis. Objectively and systematically analyze the research progress of walkability abroad from the aspects of publication overview, knowledge foundation, research direction and hot spots, etc. It is found that foreign researches on walkability mainly include three core directions: walkability and physical activity, walkability evaluation, walkability and urban design. Among them, walkability and physical activity orientation have been studied from various perspectives, such as various groups, various environmental types, different behavioral patterns and various chronic diseases. With the increasingly prominent urbanization problems and the rapid development of new technologies, multiple data, new methods and interdisciplinary cooperation will actively promote the vigorous development of walking suitability research.

## Introduction

As urbanization continues, public health issues are becoming more prominent, and in 2013 the World Health Organization (WHO) ranked physical inactivity as the fourth leading risk factor for death, noting that physical activity in China has declined by 45% over the past 18 years. At the same time, several health studies in the public health field have confirmed that walking activity can lead to many positive health outcomes, such as reducing the incidence of many chronic diseases, preventing obesity, and reducing anxiety and stress. As a result, various international health organizations have called for improving human health by optimizing urban walking environments and promoting walking activities. As early as the 1960s, scholars such as Lewis Mumford, Jane Jacobs, and William H. Whyte began to question the dominance of the automobile and advocate pedestrian-friendly urban design with a human focus. However, automobile-oriented road planning and people's over-dependence on automobiles have resulted in a pedestrian environment that has always been neglected.

The fields of public health, urban planning, and transportation planning use walkability to describe the built environment's facilitation of walking activities. Indeed, the benefits of walking for travel also include reduced traffic congestion, air pollution, improved public health environments, enhanced positive community relations, and the promotion of public transportation and enhanced parcel values. Thus, as a mode of travel, walking has the potential to simultaneously contribute to sustainable urban development in terms of health, economic, social, and environmental aspects^1^.

Research work on walkability has been carried out earlier in various fields at home and abroad, and in the past two decades, there has been a trend of a large increase in the number of studies and an update of research content in terms of theoretical foundations, research methods, research content, and the application of big data. Therefore, with the help of scientometric and network analysis methods, we can objectively and comprehensively reveal the overall publication and research dynamics of walking appropriateness, which can actively promote the development of research work in related fields in China.

## Data sources and research methods

In this paper, the Web of Science (WoS) core collection of Science Citation Index extended (SCIE) and Social Sciences Citation Index (SSCI) citation index databases were used as source databases for searching. In order to have a more comprehensive understanding of the research trends in this field, the search terms of this study are The "Topic" field also includes "walk suitability" and "walking environment". The "Topic" field was searched for topics, including articles with the above keywords in the titles, keywords and abstracts of articles in the WOS database and core journal databases in the field. The time span of the search was "1997–2019", and the types of articles were selected to represent most of the relatively complete literature of journal articles. The search yielded 1283 eligible articles, with data saved as of July 10, 2019. BibExcel, a scientometric and knowledge network analysis tool, VOSview, a visualization software, and Histcite literature analysis software were used to perform the econometric analysis and knowledge network analysis of the literature.

## Issue overview

### Analysis of the number of articles published

**Figure 1:**
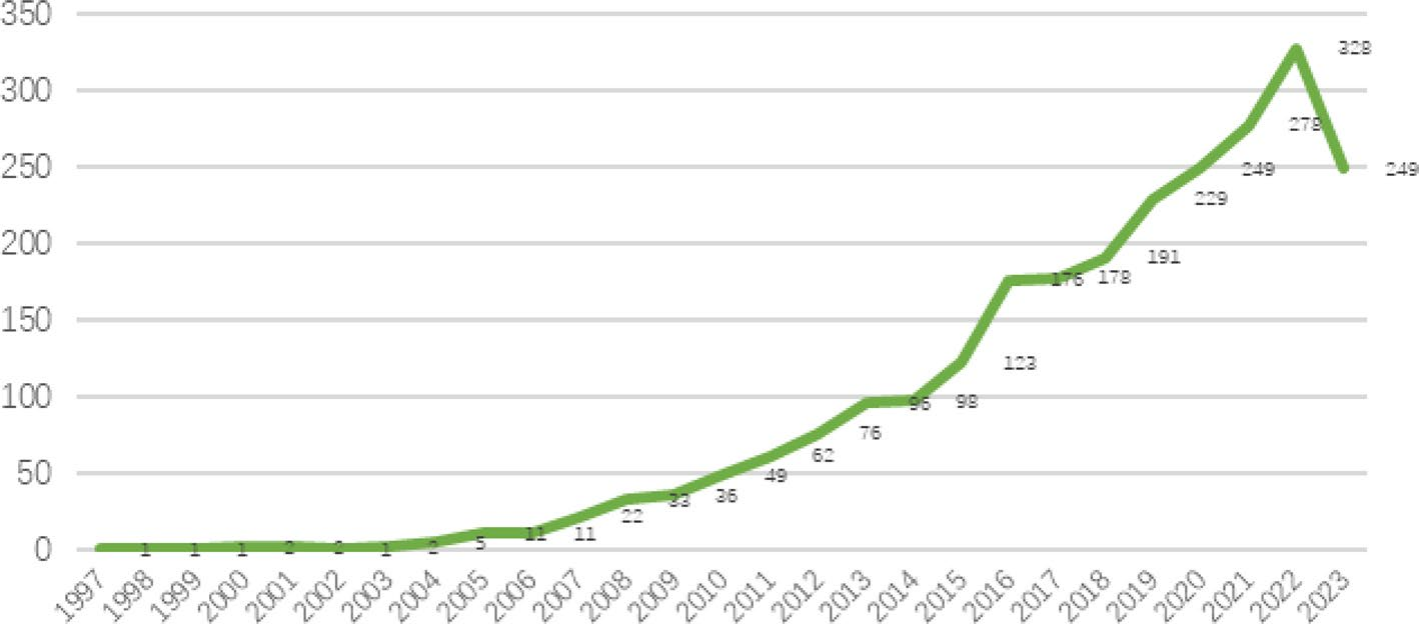
Overview of the number of articles published annually.

The change in the number of academic papers in a certain research direction is an important indicator for analyzing the development trend of that research topic. At the same time, this indicator reflects the changes in the level of knowledge in the discipline. Statistical analysis of the annual number of articles on walkability can clarify the current status of research and future trends in the field. This study summarizes the trends in the number of research papers on urban vitality from 1997 to 2018 (Fig. 1).

According to the annual article volume statistics, research on walking suitability has received increasing attention from scholars. Prior to 2004, less than 10 articles were published annually in this area of research, and since 2004, the field has been gaining attention from researchers, especially between 2014 and 2016, with a rapid increase to 191 articles per year as of 2018, and this number continues to grow. Compared with 2016, the number of articles published doubled in 2017. In addition, the number of articles published from 2019 to 2023 is almost the same as that from 1997 to 2018. This trend suggests that the field of walkability will receive more attention from researchers worldwide. The significant increase in the number of annual articles in this field in recent years may be due to the urbanization process, which has led to many urban issues such as health crisis and traffic congestion.

### Country of issue analysis

**Figure 2:**
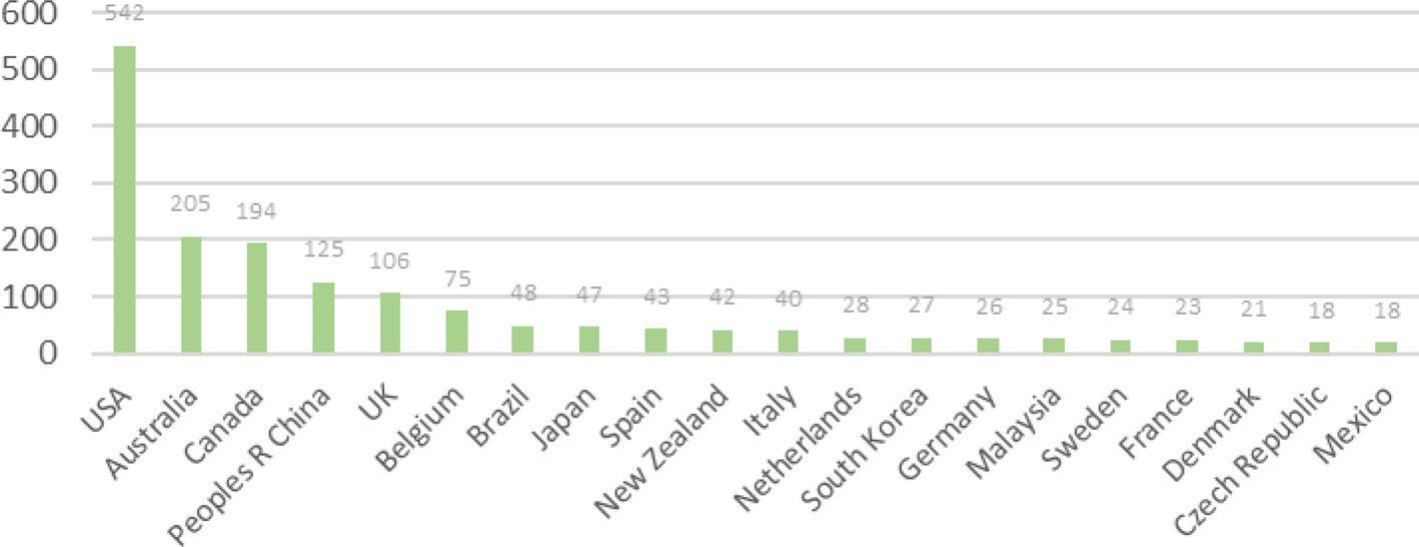
Ranking of the total number of articles issued by the country.

When the countries of publication were analyzed, all research papers on walking suitability came from 65 countries or regions in the world. Figure 2 shows the top 20 countries or regions with a total of 1677 papers, accounting for 87% of the total papers published, mainly in Europe, the Americas, Asia, and Oceania. Among them, the United States is the most active country in the study of walkability, with 542 articles, accounting for 28% of the total number of articles published, which is more than twice the number of articles published in the second place, Australia. In addition, four Asian countries, China, Japan, Korea, and Malaysia, were among the top 20 countries in this field, and China was ranked fourth in the world with 125 articles, accounting for 6.5% of the total number of articles published. It can be seen that Chinese researchers have contributed significantly to the development of walkability research worldwide.

### Keyword frequency analysis

**Figure 3:**
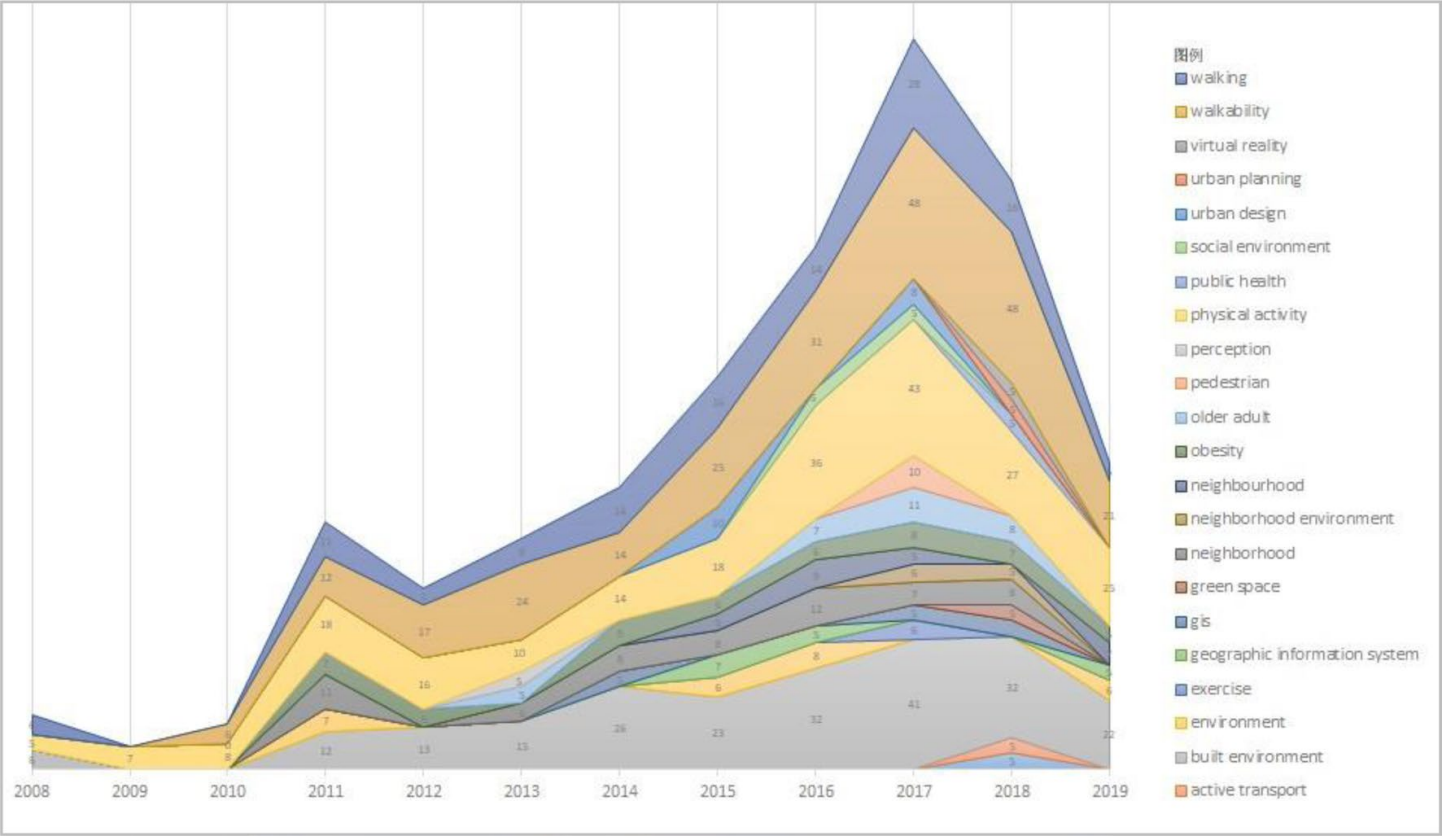
Frequency of keyword occurrence.

Studying the evolutionary dynamics of keywords can provide a quick understanding of the research trends and hot spots in the field. The study counted the key words in the field of walking suitability research in WOS from 1997 to 2019 and kept the key words with a word frequency greater than 5 per year (Fig. 3), and found that no high-frequency words appeared in the articles published before 2008, and the research content was scattered without obvious research hotspots. From 2008 onward, some key words began to appear consistently and at high frequencies in the research literature on walking suitability, e.g., physical activity has consistently been an important research content in this field since 2008. Since 2010, the number of research perspectives on walkability has been increasing, and research on the relationship between built environment, walking, obesity, elderly, community environment, urban design, and social environment and walkability has become a research hotspot in the field and has received continuous attention from researchers. In addition, the number of research papers in the direction of travel patterns, virtual reality, and big data is also on the upswing.

### Analysis of important journals

A total of 2500 articles within the search were published in 402 different publications. Among them, 199 publications have published only 1 paper in the field, and 21 journals have published more than 10 articles in all, and these journals have published a total of 1003 articles, that's more than half of the total number of articles published. This study counted the 10 most active journals among these 21 important journals (Table 1), mainly in the disciplines of public health, transportation, medicine, sports, urban planning, landscape, and sociology^2^. These journals not only have the highest contribution of publications, but also, 7 journals have the top 10 total citations of their articles, which shows that these journals are important journals in the field in terms of publication volume and academic influence.

**Table 1:** Top 10 journals by volume of published articles.

Rank	Journal	Number of published papers	Total number of citations	Average number of citations
1	International Journal Of Behavioral Nutrition And Physical Activity	105	2595	18
2	International Journal Of Environmental Research And Public Health	185	1337	78
3	Health and Place	129	2148	15
4	Preventive Medicine	82	1960	5
5	BMC Public Health	88	1073	24
6	Transportation Research Record	75	399	12
7	American Journal of Preventive Medicine	51	3998	25
8	Sustainability	181	919	17
9	Landscape and Urban Planning	67	1008	5
10	Social Science and Medicine	40	709	7

At the same time, comparing the total number of citations and the average number of citations of each journal, "International Journal of Behavioral Nutrition and Physical Activity" and "American Journal of Preventive Medicine" are the top two journals in terms of total number of citations and average number of citations. Two, the articles in this field of "American Journal of Preventive Medicine" are far more cited than those of other journals in terms of total citations or both.

## Knowledge base

**Figure 4:**
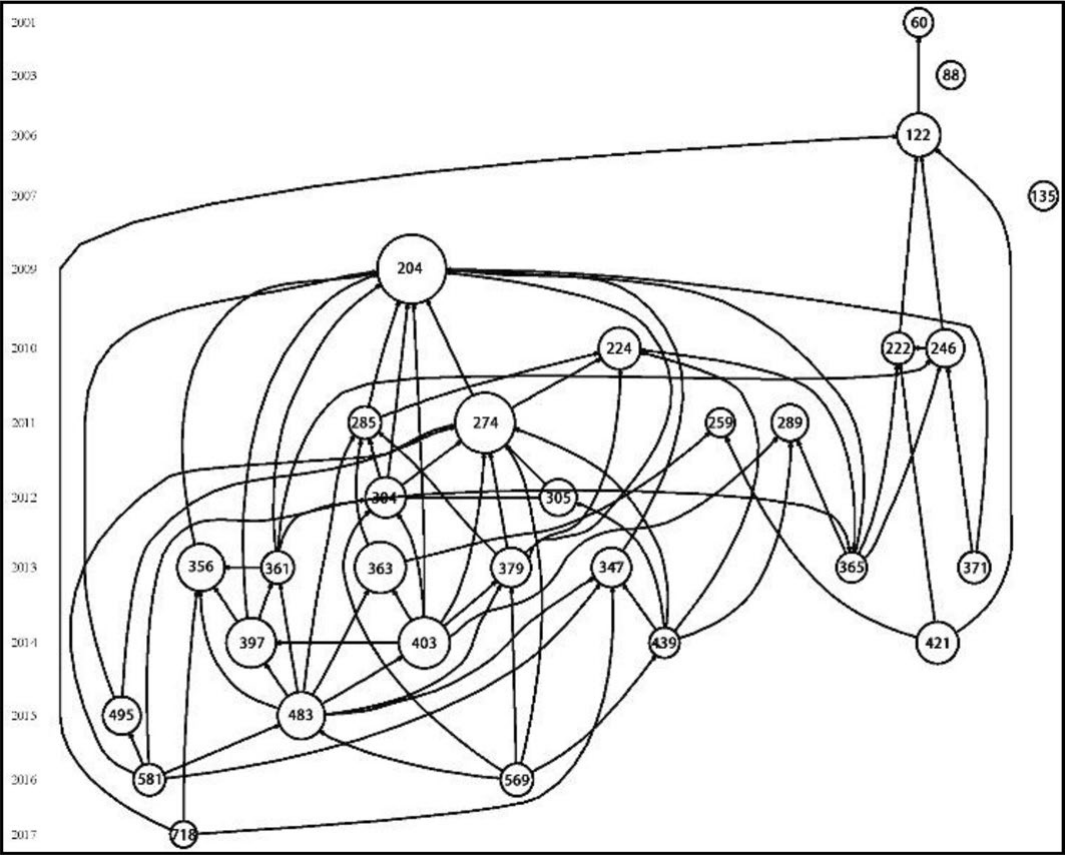
Top 30 papers in LCS and their citation relationships.

The number of articles published in each discipline was analyzed, and the top ten disciplines were mainly concentrated in the fields of public health, environmental science, transportation, and urban planning. Histcite software was used to build a database of hot literature to further analyze the most influential articles in the field of walkability, as shown in Fig. 4. The top 30 database documents with LCS scores were selected, and the 16 classic documents with more than 100 citations were analyzed. Among them, six articles focused on the potential association between built environment and walking type and physical health; four articles explored the intrinsic association between urban form, transportation, and walkability; four articles explored the related content of walkability evaluation; the remaining two articles studied the relationship between community walkability and social attributes。

## Research directions and hot spots

**Figure 5:**
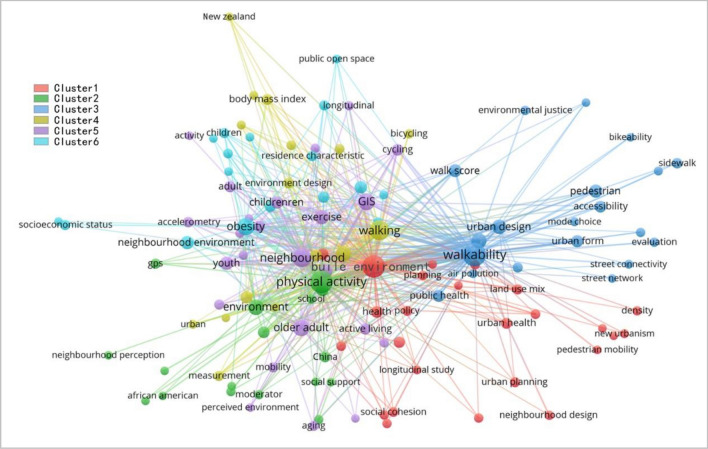
Keywords network analysis.

Keywords can express the research theme of the article, which contains the most condensed content information of the article, and network analysis of keywords can clarify the network structure and relationship strength among research directions. The keywords in the literature were extracted and analyzed by bibexcel software, and 2039 keywords without duplication were retained after data cleaning. The keywords with word frequency greater than or equal to 20 in the literature were listed and counted (Table 2), and the popular directions in the field could be found. Subsequently, the keywords with word frequency greater than or equal to 5 were network analyzed to study the intrinsic structure and association among the research directions of walk suitability. The screening results showed that a total of 121 keywords met the conditions, and then, the above keywords were expressed visually using Vosviewer software, as shown in Fig. 5, where the nodes represent keywords, and the larger the nodes indicate the higher frequency of occurrence. The thicker the line, the more frequent the co-occurrence, the higher the correlation between the keywords, and the weaker the connection strength of some keywords, which may become a new research direction in the future. Different colors represent different clusters, as shown in the figure, it can be clearly seen that the research direction of walking suitability can be divided into 6 clusters. Among them, cluster 1 is walking suitability and planning design; cluster 2 is environment type and behavior; cluster 3 is walking suitability evaluation tools; cluster 4 is the effect of physical activity on chronic diseases; cluster 5 is physical activity of different groups; and cluster 6 is walking suitability evaluation index. Some keywords with significant correlations exist in some clusters, so the six clusters are further divided into three major research directions: walking suitability and physical activity, walking suitability evaluation, and walking suitability and planning design. Finally, each research direction is associated with different regions, and the existing research directions in each region are summarized, as well as further research directions in the future (Tables 3, 4).

**Table 2:** Walkability keyword statistics.

Rank	Keyword	Keyword frequency	Rank	Keyword	Keyword frequency
1	Walkability	256	13	Exercise	34
2	Physical activity	232	14	Neighbourhood environment	29
3	Built environment	231	15	Youth	29
4	Walking	141	16	Pedestrian	27
5	Neighbourhood	111	17	Walk score	26
6	Obesity	77	18	Accelerometer	25
7	GIS	72	19	Childrenren	23
8	Older adult	64	20	Perception	22
9	Environment	55	21	Social environment	22
10	Transport	48	22	Neighbourhood walkability	21
11	Active travel	47	23	Public health	20
12	Urban design	40	24	Urban form	20

**Table 3:** Current research directions in each region.

	North America	Asia	Europe	Others
Walking environment and physical health	Child health and the built environment^26^	Neighborhood environment and physical activity^42^	Obesity and walking fitness in children^6,7,26^	The built environment and hypertension and diabetes^11^
	Obesity and the environment^8,21,22^	Some important indicators affecting walking suitability on walking^28^	School style and physical health^18^	Environmental factors affecting type 2 diabetes mellitus^24^
	Workplace environmental characteristics and walking behavior^16^		Children's gender and safety perception^4^	Community environmental characteristics and physical activity^17^
	Built environment and activity mode^22^			
	Way to school Walking environment^10^			
	Influencing factors of juvenile walking safety^5^			
Walking suitability evaluation	the relationship between four kinds of walking behavior and land use mix^29^	Urban Construction Environment Survey Tool^35^	Riding choice and environmental aesthetic factors for the elderly^30^	Evaluation of street walkability using a point system^38^
	Aesthetic rating of walking^31^	Using street pictures to assess street greening and behavior^28^	Walking fitness Index^39^	MAPS Street audit tool^34^
	Environmental pollution and walking suitability^32^		Walk index website ‘Walk Score’(Ivan Blečić et al., 2015)^40^	
	Perceived community environment NEWS or News-A series scale^36^			
	Community level aesthetic rating^31^			
Walkable and urban planning design	Root et al., 2017^31^	Built environment and walking community^41^	Environmental characteristics affecting the riding experience of the elderly^30^	Socioeconomic conditions and community walkability^44^
	Drivers of pedestrian activity^41^		Environmental quality and design tools^40^	
	Neighborhood type and walkability^42^			
	Walkability and community crime^45^			
	Community walkability and air pollution^32^

**Table 4:** Future research directions in each region.

	North America	Asia	Europe	others
Walking environment and physical health	Comparative study on data acquisition methods (e.g. self-report versus device measures)	Green space and health	A comparative study of walking environment characteristics between leisure walking and commuting walking	Geographical survey area of activity
	Improving the quality of research (e.g., using longitudinal methods, including natural experiments and newer mobile sensing technologies)	Walking environment for the elderly	The influence of urban form on physical activity	Sense the built environment and type of physical activity
	Use big data to get more accurate environmental characteristics	High-density urban walking environment	Built environment and mental health	Walking risk in the elderly
	Relationship between chronic diseases and different environmental characteristics	Walking environment and mental health	Eating behavior, built environment and obesity	More diseases and walking suitability
	The relationship between large-scale infectious diseases and urban environment	Comparative study of different cities	Adult health and the environment	
	Continue to pay attention to the relationship between the elderly and children and the built environment		Green exposure, park access and health	
Walking suitability evaluation	Take a more interdisciplinary approach	Street View images and machine learning	Study on the difference of walkability index between urban and rural areas	Multi-country comparative study
	Wider promotion of walking index	Perceptual environment and objective environment	Bicycle trip index	Pay attention to residents' perception
	More methods are incorporated into the measurement of indicators	virtual experiment	Simulation scenario study using image data and geographic data	conceptual framework
	Study on the threshold of environmental characteristics	Green space exposure	A more comprehensive assessment of urban quality	Social environment and walking environment
	Research on various types of street audit methods			
Walkable and urban planning design	Urban design and walking routes	Climate and walkability environment	A friendly city for old people and children	human-centered
	The influence of microclimate on walkability and urban design	complexity study	A multi-regional comparative study of healthy city policy	Healthy city policy
	Microscale community walkability	Region recognition method	Climate, air quality and walkability	Street service quality
	Walkability and smart city		Decision-making methods for urban renewal	Air pollution, heat island effect and walking environment

### Walk suitability and physical activity

The 2010 Toronto Charter for Physical Activity: A Global Call to Action defines the important role of physical activity in this field of study. Physical activity has multiple benefits including promoting well-being, maintaining physical and mental health, and preventing disease. Walking is a highly recommended form of physical activity as a low-impact, age-appropriate and environmentally friendly transportation choice.

#### Physical activity for different groups

Physical activity is considered to be an important intervention indicator of health level and is significantly correlated with walkability^3^. Since 2005, a large number of studies have been conducted on the physical activity patterns and environments of different populations, mainly involving children, adolescents, adults, older adults, women, and low-income groups. In children and adolescents, for example, research on physical activity and walking suitability has focused on safe walking, obesity, and school attendance. Traffic safety is an important influence on walking suitability and an important determinant of physical activity and walking to school in children and adolescents, and there is a significant correlation between perceived traffic safety and walking activity, and this association is stronger in girls than in boys^4^. And reducing traffic volume and traffic speed and increasing children's recreational areas have been shown to be important measures to increase walking safety for children and adolescents^5^. In terms of obesity prevention: physical activity can improve children's health including obesity^6^ and cardiorespiratory health^7^. Therefore, promoting physical activity in children to prevent obesity has become a public health priority^8^. Active schooling is also an important part of physical activity for children and adolescents, as well as an important means of curbing childhood obesity^9^.Yu C Y et al. found that parental tooness was a major factor in the choice of schooling, and that among the factors influencing parents to allow children and adolescents to walk to school, in addition to the amount of cars, a good environment, and walking distance, different dimensions such as safety, positive peer influence, and age of combined issues are also important influencing factors^10^. Loh et al. found that off-campus MVPA has a significant positive correlation with leisure facilities (within 2 km), walkability (1–2 km), and residential density (1 km)^11^. Ayse Ozbil et al. found that the spatial structure of the street network around the home is a decisive factor affecting children's motivation to go to school^12^.

#### Type of environment and behavior

Physical activity can be accumulated in a variety of ways, such as sports, recreation, active commuting (e.g., walking or cycling), and in different environments (e.g., schools, streets, parks, neighborhoods, etc.)^13–15^. There is consensus in the fields of urban planning, transportation, and public health that the built environment has a significant impact on whether to support residents to walk or ride. Studies have shown that people get most of their physical activity activities around workplaces, in community settings, and in walking behaviors. These built environments can create opportunities for physical activity^16^. Karen et al. studied the association between objective environmental characteristics and self-reported physical activity in 48 communities in New Zealand and showed moderately strong associations between destination accessibility, street connectivity and residential density and physical activity^17^. Robert et al. used a survey of students in Liverpool to examined the association between school travel, body mass index and cardiorespiratory health and found that active school attendance was associated with higher body mass index and lower cardiorespiratory health index, and that this conclusion was also potentially influenced by community deprivation, commuting distance, and aesthetic street characteristics^18^. Yu et al. found that lower residential density, better aesthetic environment and higher street connectivity will encourage the elderly to participate more in leisure and sports activities. Better access to services will only encourage recreational walking, rather than physical activity, among older adults^19^. Ester Cerin et al. found that complex urban environments and exposure to nature may benefit cognitive health in older adults. For higher-order cognitive abilities, such as memory, these positive effects may be stronger in areas with lower levels of traffic-related air pollution^20^.

#### Impact of built environment on chronic diseases

Lack of physical activity increases the risk of many chronic diseases, including obesity, hypertension, diabetes, colon cancer, osteoarthritis, osteoporosis, and coronary heart disease. A common consequence of physical inactivity is obesity, which as an epidemic is highly prevalent across all ages, races, and socioeconomic groups^21^. Obesity is associated with unhealthy lifestyles, education, income, and environmental factors^22^ and is associated with a variety of diseases such as cardiovascular disease, type II diabetes, and osteoarthritis^23^. Saelens BE et al. suggest that the built environment can influence energy balance by providing opportunities for physical activity, and therefore the prevalence of obesity is lower in more walkable communities than in less walkable ones^16^. Tashi et al. used data from more than 200 assessments to systematically analyze environmental factors affecting type II diabetes such as walkability, physical activity resources, food environment, green space, air to pollution, and many other environmental indicators, and the data showed that higher levels of walkability and green space were associated with a lower risk of type II diabetes, while higher levels of nitrogen dioxide, PM2.5, and noise The higher the level of nitrogen dioxide, PM2.5 and noise, the higher the risk of type II diabetes. However, due to limited data, the mechanism of environmental effects on the risk of developing type II diabetes is unclear^24^. Beulens et al., using big data samples from geographically linked electronic health records, analyzed the relationship between land use combination and intersection density in the built environment and hypertension and diabetes, and found that diabetes and hypertension were poorly controlled in African Americans. American patients are less common in more walkable neighborhoods^25^. Jia et al. followed 9440 kindergarten children from 1998 to 2007 and found that in the United States, the better the walkability of the residential area, the lower the children's body mass index and obesity risk 9 years later. This association was found in It's more pronounced among girls and suburban areas^26^. Joline W. J. Beulens et al. believe that air pollution, residential noise, and socioeconomic deprivation at the regional level are associated with increased risk of type 2 diabetes, while neighborhood walkability and green space have been associated with reduced risk of type 2 diabetes^27^ (Table 5).

**Table 5:** Finds of several studies on the built environment and health.

Example articles	Sample	Data source for environmental factor(s)	Environmental factor(s) examined	Walking metric	Results
Venurs H. Y. Loh et al.^11^	The Neighbourhood Activity in Youth study conducted among adolescents in Melbourne, Australia (n = 358, 15.3)	GIS	Recreation facilities, Park area, Walkability and walkability components (street intersections, gross dwelling density and land use mix)	MVPA outside school hours was assessed by accelerometer	Recreation facility (count within 2 km), walkability (1 km and 2 km) and residential density (1 km) had significant positive associations with MVPA outside school hours
Chia-Yuan Yu, Xuemei Zhu^10^	Students from 20 public elementary schools in Austin, Texas. (N = 2597)	Questionnaire	Whether the home–to–school distance was close enough, the presence of busy roads on the way to school, the presence of unsafe intersections, sidewalk availability, and multiple variables about the walkability (tree shade, sidewalk quality, etc.)	Walking–to/from–school behavior	Older child, positive peer influence, walkable home–to–school distance, and favorable walking environments were associatedwith more enjoyment and lower attitudinal barriers, and in turn increased likelihood of walking to/from school
Karen Witten et al.^17^	2033 adults of 48 New Zealand neighborhoods	GIS, systematic street audit	Destination access, street connectivity, dwelling density, land-use mix and streetscape quality	Light, moderate, and vigorous activity	Associations of neighborhood destination access, street connectivity, and dwelling density with self-reported and objectively measured PA were moderately strong, indicating the potential to increase PA through changes in neighborhood characteristics
Robert J. Noonan et al.^18^	194 children (107 girls), aged 9–10 years from ten primary schools in Liverpool, England	NEWS-Y	Land-use mix diversity, neighborhood recreation facilities, residential density, land-use mix-access, street connectivity, walking/cycling facilities, neighborhood aesthetics, pedestrian and road traffic safety, and crime safety	Stature, body mass, waist circumference and cardiorespiratory fitness (CRF)	Schoolchildren who lived in more deprived neighborhoods perceived by parents as being highly connected, unaesthetic and having mixed land-use were more likely to commute to school actively (p < 0.05). These children were at greatest risk of being obese and aerobically unfit (p < 0.01)
Félice Lê-Scherban et al.^25^	Data from geo-linked electronic health records. hypertension and diabetes (n = 1061 and n = 2633, respectively)	Philadelphia City Planning Commission, ESRI	Percent retail land use and intersection density	Hemoglobin A1c, blood pressure	poor diabetes and hypertension control were less common in more walkable neighborhoods
Peng Jia et al.^26^	9440 kindergarteners followed up until their 8th grade (1998–2007)	Three US national geographic datasets,GIS	Street intersection density, Residential density, Fitness facility density, recreational facility density	Children's body weight and height	In the US greater walkability in residential neighborhoods may lead to lower child BMI and obesity risk after 9 years, and the association was stronger among girls and in suburban regions

### Walk suitability evaluation

#### Walking suitability evaluation index

In the process of analyzing walkability, all urban environmental characteristics related to walking may become important indicators for assessing walkability^3^. Existing research results, on indicators of walkability, are divided into two main dimensions: (1) to determine evaluation indicators from urban scale overlooking density, diversity, and design standards to quantitatively describe the overall urban factors affecting walkability. For example, Lu et al., studied the linkage between pedestrian suitability and urban-scale environmental influences in Hong Kong by using population density, land use mix, and street connectivity as evaluation indicators, and found that two indicators, population density and land use mix, were not significantly correlated with any area of walking, while population density was positively correlated with traffic walking and recreational walking only in the lower density range, and in the higher The higher density range was negatively correlated with recreational walking, therefore, there may be no effect on walkability in high-density cities using the above indicators^28^. Lisa et al. surveyed adults in the suburban subway neighborhoods of Vancouver to assess the association between the four walking behaviors of walking to work, walking out, walking for recreation, and physical activity and land use mix and found that living in areas with a high proportion of commercial land, recreational areas with a high proportion of land use increases the likelihood of walking out; living in areas with a low land use composition may lead to low levels of recreational walking^29^. (2) Examining cities at the street and community scale, and using evaluation indicators for safety, environmental quality, attractiveness, aesthetics, and comfort to qualitatively analyze walkability.Van Cauwenberg et al. analyzed seven environmental themes such as traffic safety, infrastructure, road maintenance, connectivity, and aesthetics that may affect the cycling experience of older adults, and concluded that traffic safety is an important factor influencing older adults' cycling choices^30^. Root E D et al. conducted an empirical study of aesthetic ratings of community walking and used spatial regression models to find that aesthetic ratings were influenced by geography, aesthetic perception, and race^31^. Besides, some relevant factors such as socioeconomic status and air pollution are also important indicators in some walkability studies. For example, Marshall et al. monitored the concentrations of carbon monoxide and ozone in the air in some areas of Vancouver and studied the association between environmental pollution and walkability, and found that areas with low pollution and high walkability are usually located near downtown and are almost always high-income communities^32^.

#### Walk suitability evaluation tool

The influence of environmental factors on walking has led to a large number of tools to measure environmental walkability. Walkability tools were developed to assess the overall built environment and to help identify and clarify the factors that impede walkability^33^. Currently, research on walkability assessment tools has focused on two components: audit tools and analytical tools. The use of environmental audit tools is mainly to investigate the performance, spatial demand, and walking experience of pedestrian facilities through GIS measurements, questionnaires, and direct observation^34^. For example, the China Urban Built Environment Survey Tool (CUBEST) developed items including six dimensions of residential density, street connectivity, accessibility, sidewalk quality, bicycle lane quality, and environmental aesthetics, and professional investigators rated the environment based on the survey items in the above dimensions^35^; a series of scales such as NEWS or NEWS-a for perceived community environment research, which has been used by researchers in multiple countries as an audit tool for walkability studies to investigate residents' perceived community environment^36^; and analytical tools such as scoring systems, simulations, regression analysis, and integral logit models are mainly applied in the evaluation of environmental walkability^37^. For example: Root et al. investigated the factors of community-level aesthetic scores, applied spatial regression models, and the introduced metric of spatial autocorrelation, and verified through empirical analysis that socio-environmental, economic, and spatial structure aspects play an important role in shaping community aesthetics, providing health and planning personnel with ways in which interventions can be taken^31^; Gallin et al. used a point system to assess the pedestrian suitability of streets, which provides a good basis for evaluating the level of service of pedestrian facilities by rating the surveyed streets with geographic information data and field research assessments^38^. In addition to this, the number of articles on pedestrian suitability evaluation using a composite index is on the rise. The composite index is a summation weighting of the indicators that predict a representative environment that is conducive to walking. As the composite index of walkability has gradually entered more fields such as urban economy, transportation, and real estate, it has transcended disciplinary boundaries to form walkability indices based on different evaluation indicators with specificity. For example, D'Alessandro D et al. proposed the walkability index (T-WSI) measurement method, which uses direct observation of utility, safety, urbanity, and pleasantness, four categories of 12 weighted indicators, to form a comprehensive walkability index and identify streets and elements with low walkability index to support community walkability optimization strategies proposed^39^. With the advancement of geospatial technology and the availability of online maps and datasets, several research teams have developed online assessment tools. For example, the Walk Score, a walkability index website, uses publicly available data (population density, intersection density, block length, etc.) to quickly and freely calculate and score the walkability of an address to nearby amenities, addressing the time-consuming nature of common measurement studies; the Walkshed website has derived a walkability The Walkshed website has spawned a walkability surface tool that assesses the quality and diversity of unimpeded access to an address within a 1-mile radius and calculates a walkability heat map by prioritizing a set of factors by the user; in addition, such walkability tools include "Walkonomics", "WalkYourPlace", and "WalkYourPlace", "WalkYourPlace", etc.^40^. The development of online assessment tools offers a broader prospect for the flourishing of walking suitability-related research.

### Pedestrian suitability and planning design

An important purpose of the study of walkability in urban planning is to link it to urban planning and design. Locations with higher walkability tend to have more active residents and are conducive to a healthy urban life. Walkability is often related to a variety of factors, and the results of this research have contributed to the weighing of pros and cons in the planning and design process, as well as to policy development.

Population density, land use diversity, and street connectivity are considered to be important indicators of walkability. Yi Lu et al. used the above variables to investigate the correlation between pedestrian commuting and recreational walking in Hong Kong and found significantly different results; land use diversity and street connectivity were not significantly correlated with the two types of walking trips, and population density was only positively correlated with pedestrian commuting and recreational walking were positively correlated in the higher density range, and negatively correlated with recreational walking in the higher density range^28^; Lai et al. explored the drivers of pedestrian activity in cities by integrating multiple datasets on pedestrians and the environment in cities and quantifying place location characteristics. It was found that building density, population density, and public transportation were persistent drivers of pedestrian activity in New York City, while other features of the urban landscape influenced pedestrian activity differently depending on time of day, day of week, and surrounding environment^41^. Zuniga-Teran et al. compared the walkability of four types of communities: traditional, suburban, gated, and clustered communities and found that Traditional communities were the only ones that differed from other types of communities and were friendly to both traffic and recreational walking, but also had the disadvantage of higher crime rates, while enhancing the natural environment and regular maintenance of the community may improve the well-being of residents, regardless of the type of community^42^. As research progresses, some pedestrian suitability evaluation models can be used not only as analytical tools, but also as support systems for planning and design. For example, Ivan Blečić et al. proposed a planning and design software tool, Walk Explorer (WE), which can synthesize the actual routes of community walkability and walking-related environmental qualities, and use a multi-criteria evaluation model as the core of a decision support system to score the walkability of various points in urban space and derive potential walking routes along the street network; it can also be used as a tool to improve the effectiveness, relevance, and inclusiveness of urban design and tool for improving the effectiveness, relevance, and inclusiveness of transportation planning^40^. Jin Rui et al. proposed that measures related to innovative streets can be divided into two broad categories: urban mobility and urban livability. Combining smart street facilities with the Internet of Things (IoT) using grid and radial street networks to create a safe street environment was found to be critical to promoting urban mobility. In contrast, walkable, cycle-friendly and people-centered street environments enhance social interaction and urban livability^43^.

In addition to this, traffic, weather, safety, economic, and psychosocial factors have been shown to be important factors influencing walkability to some extent, affecting the nature and extent of walkability^44^. For example, Sarah Foster et al. studied the correlation between walkability and community crime and found that crime rates were positively associated with walkability and that more walkable neighborhoods also had the potential to attract higher levels of crime, and therefore the impact of crime rates also needed to be weighed in planning and design^45^; Julian et al. studied the correlation between community walkability and air pollution and found that different urban spatial patterns result in different concentrations of ozone and that low-income areas have both high and low walkability characteristics and higher levels of both motor vehicle tailpipe pollution and ozone pollution. In contrast, high-income areas are generally located near city centers and have low pollution and high walkability characteristics. This shows that the increasing concentration of urban layout and activities can bring both better walkability and air pollution disadvantages, and therefore, more external factors of the built environment need to be incorporated into the planning and design for comprehensive consideration^32^.

## Summary and discussion

This paper uses scientometrics as the theoretical basis for data analysis and combines knowledge network analysis methods to conduct knowledge mapping analysis of literature published in the Web of Science database from 1997 to 2019, which visually presents the overall publication situation of the existing literature in walking suitability-related research. With more and more research results confirming that physical health and sustainable development are closely related to the walking environment, the research on walking suitability has received increasing attention and the number of publications has increased significantly. The research content is gradually diversified and refined, incorporating multidisciplinary knowledge from public health, urban planning, transportation, behavioral science, geography, etc., and gradually penetrating into research with different populations, different behaviors, different types of environments, multidimensional indicators, and multiple data. Research methods have also shifted from early research and qualitative analysis to a comprehensive research approach that combines multiple data, constructs assessment models, and combines qualitative and quantitative, static and dynamic. Due to the accelerated urbanization and the increasing health problems, the research directions related to the evaluation of walkability, walkability and physical activity, and walkability and planning and design will continue to be the focus of attention in the future. In terms of walking environment and physical activity, researchers mainly focus on several key directions, including: different groups and physical activities, environmental types and behavioral patterns, and the impact of the built environment on chronic diseases. Research results in various regions mainly focus on the impact of the built environment on walking activities and travel patterns of various people. North America and Europe have also conducted a large number of studies on the impact of the built environment on various chronic diseases such as obesity and diabetes. In future research, various. The region will continue to pay attention to the impact of the built environment on chronic diseases and infectious diseases, and the impact of the built environment on children and the elderly. Researchers in North America will also focus on how to use different types of data and different research methods to improve the quality and accuracy of research. Sexually speaking; Asia is affected by dense population and development stage. The built environment and mental health, green exposure and physical activity, and pedestrian environment design in the urban renewal process will also be key areas of future research. In terms of walking suitability evaluation, walking suitability evaluation indicators and walking suitability evaluation tools are two important research directions. Currently, various regions are focusing their research on developing various street audit tools to formulate a system for different groups of people, different environments, and Various evaluation indicators are used in different ways, and many researchers have developed different evaluation tools for walkability; North America will explore the inclusion of more methods and more interdisciplinary research into research in this field, and explore more accurate indicator threshold; Asia will continue to focus on research on perceived environment and objective environment, street view maps and machine learning; Europe will conduct in-depth research on urban quality evaluation and spatial simulation using multi-source big data. In terms of walkability and urban design, it mainly discusses how to apply the research results in this field to the urban design process. The impact of air quality, climate and other aspects on the built-up area and pedestrian environment is a continuing concern of researchers around the world. Research directions, in addition to smart cities, micro-communities and how walkability guides urban policies, are also very valuable research directions in this field.

Based on the above research, the following are some suggestions for the future research on walkability in China: (1) Combine the national conditions of China and reasonably draw on the research results from home and abroad. There are many inconsistencies in the results of research on walkability, which may be related to the acquisition of research data, interpretation of analysis results, and cultural differences between cities. (2) Expand the depth and breadth of research. Although the research on walking suitability in China has been carried out late, there have been many promising results, including studies on walking suitability evaluation, street vitality, physical activity, public transportation, etc., which have accumulated valuable information for the development of walking suitability research. However, it is still found that the research results in China lack richness in terms of research content and research methods, with more case studies and fewer empirical studies. Researchers still need to combine the actual situation of urban development and search for research content and perspectives among the relevant theories and achievements in the fields of public health, urban planning and behavioral science to meet the needs of urban development. (3) Strengthen interdisciplinary cooperation and application of new technologies. China's cities face various problems such as traffic congestion, health risks, and air pollution, and research related to walkability needs to seek interdisciplinary complementary advantages and expand new ideas and achievements. Meanwhile, the development of big data, geographic information technology, and artificial intelligence has provided new algorithms and data sources for urban environment measurement, human behavior activity monitoring, and walking suitability definition, creating new perspectives and ways of thinking for researchers, and the application of new technologies for walking suitability research has become the frontier of research in this field and has provided the possibility for its continuous development.

## Data Availability

The data used to support the findings of this study are all in the manuscript.
